# Signaling Pathways Driving Aberrant Splicing in Cancer Cells

**DOI:** 10.3390/genes9010009

**Published:** 2017-12-29

**Authors:** Vânia Gonçalves, Joana F. S. Pereira, Peter Jordan

**Affiliations:** 1 Department of Human Genetics, Instituto Nacional de Saúde Doutor Ricardo Jorge, Avenida Padre Cruz, 1649-016 Lisboa, Portugal; vania.goncalves@insa.min-saude.pt (V.G.); joana.pereira@insa.min-saude.pt (J.F.S.P.); 2 BioISI—Biosystems & Integrative Sciences Institute, Faculty of Sciences, University of Lisbon, 1649-004 Lisboa, Portugal

**Keywords:** alternative splicing, cancer cell, genetic program, signaling pathway, tumorigenesis, tumor microenvironment

## Abstract

Aberrant profiles of pre-mRNA splicing are frequently observed in cancer. At the molecular level, an altered profile results from a complex interplay between chromatin modifications, the transcriptional elongation rate of RNA polymerase, and effective binding of the spliceosome to the generated transcripts. Key players in this interplay are regulatory splicing factors (SFs) that bind to gene-specific splice-regulatory sequence elements. Although mutations in genes of some SFs were described, a major driver of aberrant splicing profiles is oncogenic signal transduction pathways. Signaling can affect either the transcriptional expression levels of SFs or the post-translational modification of SF proteins, and both modulate the ratio of nuclear versus cytoplasmic SFs in a given cell. Here, we will review currently known mechanisms by which cancer cell signaling, including the mitogen-activated protein kinases (MAPK), phosphatidylinositol 3 (PI3)-kinase pathway (PI3K) and wingless (Wnt) pathways but also signals from the tumor microenvironment, modulate the activity or subcellular localization of the Ser/Arg rich (SR) proteins and heterogeneous nuclear ribonucleoproteins (hnRNPs) families of SFs.

## 1. Introduction

Transcription of a protein-coding gene in human cells generates a primary transcript, the pre-mRNA that still contains intervening intronic sequences, which in general are co-transcriptionally removed during the process of mRNA splicing. This process is catalyzed by the spliceosome, a macromolecular machinery involving five small nuclear ribonucleoprotein particles (U1, U2, U4, U5, and U6 snRNP), which recognizes conserved nucleotide sequences around each exon-intron junction [1,2]. Additional sequence elements in exons or introns can act as enhancers or silencers, and mediate the binding of splicing factors that either promote or inhibit productive recognition of a given exon by the spliceosome. Two main families of such splicing factors are the Ser/Arg rich (SR) proteins and the heterogeneous nuclear ribonucleoproteins (hnRNPs), which often act antagonistically and thus allow regulation of exon recognition through a combinatorial mode of operation. 

Eukaryotic cells use this processing step to increase their diversity in gene expression by generating various mRNA isoforms from the same pre-mRNA through alternative splicing. The era of genome-wide high-throughput sequencing technologies has revealed that over 90% of human genes can undergo alternative splicing [3,4,5,6,7]. Most frequently observed is the inclusion or skipping of individual exons, but other variations include the use of alternative 5′ or 3′ splice sites within exons, intron retention, or alternative terminal exons with distinct polyadenylation sites. In addition, the usage of alternative promoters can contribute with different first exons to pre-mRNAs transcribed from the same gene [8].

From a functional point of view, an alternative spliced transcript can have two effects: (i) either it is translated into a protein with different functional properties [9,10,11], (ii) or it regulates the respective gene’s expression level. Examples are the degradation of nuclear-retained intron-containing transcripts [12], the degradation of premature stop codon containing alternative transcripts by the nonsense mediated decay pathway [13,14], or the modulation of mRNA stability or translation efficacy by loss of microRNA (miRNA) binding sites in alternative 3′-untranslated regions [15,16]. 

Different tissues share the same genome but generate different profiles of alternative splice variants as part of their gene expression programs [17,18,19,20]. Since tumor development is also characterized by gene expression program changes, aberrant profiles of alternative splicing were consistently reported [21,22,23]. In contrast to genetic diseases, where a disease-causing gene can suffer a mutation that interferes with the normal splicing process of its mRNA, aberrant profiles observed in tumors mostly reflect the selection of endogenous alternative splicing variants with different functional properties that allow the malignant progression of initiated tumor cells. Selected functions relate, for example, to sustained proliferation, evasion of apoptosis, metabolic adaptation, or angiogenesis. Thus, the observed changes are not simply bystander effects of altered cellular programs but contribute specifically to tumor progression. Some well-studied examples of such alternative splicing variants are described in this review and have been summarized in Supplementary Table S1.

The aberrant splicing patterns observed in cancer can originate from different molecular mechanisms. Whereas mutations in splicing-regulatory sequences of target genes are frequently detected in genetic diseases, this does not seem to be the major event during sporadic tumor development. It should, however, be mentioned that the application of next-generation sequencing technologies has detected cases with gain-of-function point-mutations in splicing factor genes, mainly the constitutive spliceosome factors, splicing factor 3b subunit 1 (SF3B1) and U2 small nuclear RNA auxiliary factor 1 (U2AF1), in myeloid cell malignancies and rare cases of breast or lung cancer [24,25,26]. Another target are inactivating mutations in zinc finger CCCH-type, RNA binding motif and serine/arginine rich 2 (*ZRSR2*) gene encoding a component of the minor U12 dependent-spliceosome that result in a variety of alternative splicing variants related to oncogenic signaling pathways [27]. Thus, it cannot be excluded that certain mutations may exert a driver effect in tumorigenesis by altering signaling pathway dynamics.

More consistently reported was that tumors display changes in splicing factor expression levels [28,29,30,31]. These changes disturb the balance between cooperating or antagonizing splicing factors in genome-wide splicing decisions, and can exert a clear oncogenic effect, as documented for splicing factor serine and arginine rich splicing factor 1 (SRSF1) [32]. 

Two aspects need to be considered in this respect: transcriptional changes in the expression of splicing factor genes and the post-translational modification of the corresponding splicing factor proteins. The genetic alterations underlying the observed changes in splicing factor expression in tumors remain mostly unclear; however, recent genome-wide analyses have emphasized the existence of a ‘dark matter’ in cancer genomes that requires better characterization, namely the potential cancer-driving mutations in non-coding regions that may affect gene regulation steps such as transcription, splicing, mRNA stability, or translation [33]. Another mechanism disturbing the balance is the post-translational modification of splicing factor proteins, with consequences for their activity, subcellular localization, or proteasomal degradation. Significant progress has been made in understanding these more dynamic aspects of splicing regulation. Therefore, this review will focus on the deregulation of splicing profiles in tumor cells in consequence of either oncogene-driven signaling or signals received from the tumor microenvironment.

## 2. Deregulation of Alternative Splicing by Oncogene-Driven Signaling

Our understanding of tumor cell biology is intimately connected to the determination of certain hallmark capabilities, which include self-sufficiency in proliferative signals and evasion of growth suppressing mechanisms [34]. Most of these capabilities are accomplished through signaling pathways that are typically composed of a signal-perceiving receptor, which transmits successful ligand binding into a chain of biochemical events among a group of molecules inside the cell to control a cell function or response. The biochemical events involve protein interactions and post-translational protein modifications, most commonly protein phosphorylation catalyzed by protein kinases. Abnormal activation of such signaling pathways is common in cancer and provides attractive targets for drug development to block the growth and survival of malignant cells [35].

Abnormal activation originates in the cancer cell from gene mutations that either result in growth factor receptor activation in the absence of their ligand, or activate downstream components of growth factor receptor-stimulated signaling cascades, or eliminate factors conferring negative feedback regulation. These are cancer cell intrinsic alterations and were found to clearly affect alternative splicing decisions, as will be described in the following sections.

### 2.1. Oncogenic Stimulation of the RAS/RAF/ERK Pathway

Activation of the small GTPase RAS with subsequent stimulation of a cascade of three mitogen-activated protein kinases (MAPK, namely RAF, MEK and ERK), is a key event in the majority of epithelia cell-derived tumors. Oncogenic Kirsten rat sarcoma viral (KRAS) mutations that activate the pathway in the absence of ligand binding to plasma membrane receptor tyrosine kinases are a hallmark of many epithelial cancers. Bioinformatic analysis of transcriptomic data obtained from colon adenocarcinoma or lung squamous cell carcinoma revealed that the presence of oncogenic KRAS correlated with a regulatory network of functional splicing aberrations. The network involved increased levels of the transcription factors ETS transcription factor (ELK1) and avian myelocytomatosis viral oncogene homolog (MYC), which both together then induced expression of the alternative splicing factor polypyrimidine tract-binding protein 1 (PTBP1). Increased PTBP1 levels associated with a shift in the alternative splicing of transcripts encoding the small GTPase RAC1, adaptor protein NUMB, and Pyruvate Kinase (PKM), all of which have an experimentally documented role in tumorigenesis [36]. The PTBP1 overexpression was detected in colorectal cancer tissues when compared with corresponding normal mucosa, and correlated with increased c-MYC expression levels and altered ratios between the two alternative PKM variants, PKM2 and PKM1 [37], which contain the mutually exclusive exons 10 or 9, respectively, and differ in enzyme kinetics. In cancer cells, PKM2 expression promotes the accumulation of upstream glycolytic intermediates to fuel the anabolic metabolism. 

Besides transcriptional stimulation, the activation of the MAPK extracellular signal regulated kinase (ERK) downstream of RAS can also lead to the direction of the phosphorylation of splicing factors. For example, the inclusion of exon v5 into the cell surface tumor marker clusters of differentiation 44 (CD44) enhances malignancy and invasiveness of some tumors, and is regulated by splicing factor signal transduction and activation of RNA metabolism 68 (SAM68). ERK phosphorylates SAM68 in T-lymphoma cells [38,39], enhancing its RNA binding activity. The same pathway was described in colorectal cancer cells and leads to binding of phospho-SAM68 to the 3′UTR of the *SRSF1* transcript [40]. The binding promotes retention of an intron required for production of full-length *SRSF1* transcript and avoids an alternative splicing event that would downregulate *SRSF1* transcripts through the nonsense-mediated mRNA decay pathway. Thus, ERK activation results in an increased level of SRSF1 protein and corresponding switch in the splicing profile, e.g., of the *RON* gene transcripts (see Section 3.1.1).

Furthermore, ERK2 phosphorylates splicing factor SPF45 (RBM 17) in cells carrying oncogenic Harvey rat sarcoma viral oncogene homolog (HRAS)^G12V^ or B-isoform of rapidly accelerated fibrosarcoma (BRAF)^V600E^ mutations. Phosphorylation of SPF45 led to the inclusion of the alternative extra domain I (ED-I) region into fibronectin transcripts, which increases the ability to promote cell cycle progression and wound healing, but also to exon 6 exclusion from First apoptosis signal (*FAS)* mRNA generating a variant that inhibits FAS-mediated cell death [41]. Interestingly, SPF45 was shown to be also phosphorylated by Jun N-terminal kinase 1 (JNK1) and p38α MAPK, suggesting it can be modulated through multiple pathways.

Another mechanism known to affect alternative splicing is nucleocytoplasmic distribution of splicing factors. In colon cancers carrying an activating mutation in the RAS-interacting kinase BRAF, RANBP2 was found to be upregulated [42]. This protein regulates nucleocytoplasmic transport of phosphorylated pre-mRNA processing factors as well as their speckled intra-nuclear distribution, and RANBP2 overexpression affected alternative splicing patterns, including the transcripts encoding CDC-like kinase (CLK) and the anti-apoptotic splice variant BCL-x(L) of apoptosis regulator *BCL2L1* [43].

Interestingly, a recent paper suggested that hnRNPA2 up-regulation in hepatocellular carcinoma induces an alternative splicing switch that downregulates a dominant-negative isoform of A-RAF, leading to activation of the RAF-MEK-ERK pathway and cellular transformation [44]. This example highlights that the generation of certain alternative splicing variants can also shape cancer cell signaling, for example by exploiting feedback mechanisms that further sustain tumor cell survival.

### 2.2. Stimulation of the PI3K/AKT Pathway 

The phosphatidylinositol 3 (PI3)-kinase pathway (PI3K) is another key pathway involved in cell survival and escape from apoptosis in a significant number of solid tumors. It involves the phosphorylation of phospholipid components of cell membranes that then serve as docking sites for the recruitment of proteins to the plasma membrane, and subsequent activation of signaling cascades. This pathway is mainly activated by mutation or amplification of the PI3K catalytic subunit α (*PIK3CA)* gene, or inactivation of the antagonistic phosphatase and tensin homolog *(PTEN)* phosphatase gene [45,46]. The effector following activation of this pathway is protein kinase v-akt murine thymoma viral oncogene homolog (AKT), which triggers the phosphorylation of splicing regulatory proteins. For example, AKT was shown to phosphorylate SRSF1 and SRSF7 proteins in vitro when immunoprecipitated from HEK293T or COS-7 cells [47], and overexpression of constitutively active AKT increased alternative splicing of a cell cycle promoting fibronectin transcript. A subsequent study raised the possibility that the described abilities of immunopurified AKT to phosphorylate SR proteins may be caused by associated SRSF protein kinase (SRPKs), because in epidermal growth factor (EGF)-stimulated HEK293T cells AKT1 promoted SRPK1 and SRPK2 autophosphorylation and translocation to the nucleus, with corresponding changes in alternative splicing of an Adenovirus early region 1A (*E1A)* minigene reporter [48]. On the other hand, another arginine-serine rich (RS) domain-containing protein, the Lamin B receptor, was shown to be directly phosphorylated by both AKT1 and SRPK1 [49]. The SRPK1 activity is important for binding SR proteins with high affinity in the cytosol and progressively phosphorylating 10–12 serine residues in the N-terminal region of the RS domain (RS1). This modification is required for nuclear import of SR proteins and their typical localization in speckles [50,51].

Similar splicing modulation was found for caspase-9 (Casp-9) in lung cancer cells, in which activated AKT phosphorylated SRSF1, thereby leading to exclusion of an exon 3,4,5,6 cassette and generation of the anti-apoptotic Casp-9b isoform [52]. In parallel, AKT-mediated phosphorylation of hnRNPL induced its binding to a splice silencer element in Casp-9 pre-mRNA, further enhancing the exclusion of the above mentioned four-exon cassette [53,54]. 

The PI3K/AKT pathway is also known to activate the mammalian target of rapamycin complex 1 (mTORC1), a key regulator of cell metabolism and growth. It controls the rate of protein synthesis via the eukaryotic translation initiation factor 4E-binding protein (4E-BP) and ribosomal protein S6 kinase (S6K), and is inhibited by the anti-cancer drug rapamycin [55]. Recent data revealed an mTORC1-S6K1 pathway leading to phosphorylation of kinase SRPK2, which translocates to the nucleus and activates SR protein binding to the U1-70K spliceosome component to promote splicing of lipogenesis-related transcripts to fuel cancer metabolism [56].

In hepatocellular carcinoma, RAS signaling led to AKT activation and subsequent SRSF1-dependent splicing of the SV1 isoform of Krüppel-like factor 6 (KLF6), a cytoplasmic inactive variant of this tumor-suppressing transcription factor [57]. Also, phosphorylation of SRSF5 by AKT2 has been shown to regulate exon inclusion in the protein kinase C (PKC) βII isoform [58,59], which is associated with colon carcinogenesis [60].

### 2.3. Stimulation of the Wnt Pathway 

The wingless (Wnt) signaling is important in regulating development and stemness, and its tight association with many cancer types has been well studied in colorectal cancer (CRC) [61]. Upon Wnt ligand binding, the transcriptional co-activator β-catenin is protected from proteolytic degradation, which is otherwise triggered by a multi-protein destruction complex comprising Axin, Adenomatous polyposis coli (APC) protein and protein kinase glycogen synthase 3β (GSK3β). Crosstalk between Wnt signaling and alternative RNA splicing in CRC includes effects on the expression of Ras-related C3 botulinum toxin substrate 1b (RAC1b), an alternatively spliced variant promoting nuclear factor of κ light polypeptide gene enhancer in B cells (NF-κB) activation and tumor cell survival. Activated β-catenin signaling directly increased the transcription of the *SRSF3* gene [62]. One described effect of SRSF3 is the negative regulation of *RAC1* exon 3b inclusion and thus of RAC1b levels [63] that are upregulated in *BRAF*-mutant CRC [64]. 

By contrast, inclusion of *RAC1* exon 3b was shown to require SRSF1 in colorectal cells [63] and more recent studies revealed that protein kinases SRPK1 and GSK3β act upstream of SRSF1 [65]. SRPK1 and SRSF1 also mediated alternative splicing of two mutually exclusive exons 4A and 4B of the solute carrier family 39 (zinc transporter) member 14 (*SLC39A14*) gene, encoding a metal ion transporter. In colorectal tumors, the Wnt pathway induced expression of a high-affinity variant [66]. GSK3β is part of the canonical Wnt signaling pathway, but can also be regulated through an inhibitory phosphorylation by AKT. Surprisingly, this mechanism is apparently not used for regulating RAC1b in colorectal cells because only GSK3β, but not AKT depletion, affected RAC1b transcript levels [65]. A role for GSK3β in regulating splicing has also been reported through direct phosphorylation of splicing factors such as SRSF2 [67] or PTB-associated splicing factor (PSF) [68], and can further be deduced from the lack of phosphorylation of RNA splicing factors (including SRSF9, Serine and arginine repetitive matrix 1 (SRRM1); SRRM2; Transformer 2 β homolog (TRA2B); SRSF10; and CUGBP elay-like family member 1 (CELF1)) in GSK3 knock-out cells, which was associated with 194 splicing differences in 188 genes [69]. 

Another important splicing factor controlled by the Wnt signaling in colon cancer cells is PTBP1. Its gene expression is controlled by a transcriptional complex formed by β-catenin, T Cell-specific transcription factor/Lymphoid enhancer-binding factor (TCF/LEF) and nuclear phospho-PKM2 (pSer37), a phosphorylation performed by ERK in response to KRAS activation [70].

### 2.4. Other Pathways Activated in Proliferating Cells

The soluble second messenger cyclic adenosine monophosphate (cAMP) is generated following activation of G-protein-coupled membrane receptors by hormones or local mediators and activates protein kinase A (PKA), which is involved in the regulation of cell proliferation during the onset and progression of various tumors [71]. Splicing regulator PTBP1 was found to be directly phosphorylated on Ser-16 by PKA, resulting in the accumulation of PTBP1 in the cytoplasm [72]. 

Another PKA regulated protein is transcription factor Wilms tumor 1 (WT1) that binds to and represses the promoter of the *SRPK1* gene through a specific WT1 binding site. Phosphorylation of WT1 on Ser 365 and 393 blocks its DNA binding and results in its cytoplasmic retention [73,74]. In the absence of functional WT1, SRPK1 expression and subsequent hyperphosphorylation of SRSF1 increased, promoting, for example, the expression of the pro-angiogenic vascular endothelial growth factor (VEGF) splice variant VEGF165 [75].

Casein kinase 2 (CK2) is a further protein kinase involved in the regulation of SRPK1. Biochemical evidence in testis extracts showed phosphorylation of SRPK1 by CK2 at Ser51 and Ser555, increasing its enzymatic activity in vitro [76]. Elevated levels of protein kinase CK2 have long been associated with increased cell growth and proliferation both in normal and cancer cells [77].

Calcium signaling is yet another pathway contributing to tumor progression, mainly via metabolic adaptation of tumor cells or their evasion from apoptosis [78,79]. An influx of Ca^2+^ ions was shown to trigger activation of calcium/calmodulin-dependent protein kinase type IV (CaMKIV), which phosphorylates the splicing repressor hnRNPL and enhances its binding to an RNA element called the CaRRE motif near the 3′ splice site of several target gene transcripts. This modulates alternative splicing events, including the skipping of the stress axis regulated exon (STREX) of potassium channel *SLO1* that confers higher channel activity [80,81]. 

## 3. Deregulation of Alternative Splicing by Tumor Microenvironment-Derived Signaling

Although cancer cells accumulate mutations and genetic changes during tumor progression, recent advances have suggested that this alone may not be sufficient to drive cancer as a clinical disease [82]. The tissue microenvironment provides crucial signaling to initiated tumor cells. When intact, it can inhibit the growth of existing malignant cells and maintain tissue architecture. On the contrary, a microenvironment characterized by chronic inflammation, oxidative stress or immune-suppression will promote tumor cell expansion. A dynamic and reciprocal interaction between the neoplastic and stromal cells exists along the stages of tumorigenesis. In the following, microenvironmental stimuli such as stromal cell-derived soluble factors or the extracellular matrix are reviewed that were identified to induce changes in alternative splicing of epithelial tumor cells. 

### 3.1. Soluble Factors

#### 3.1.1. Growth Factor Signaling

Under physiological conditions, cells receive fate-determining signals from their tissue surroundings, primarily in the form of polypeptide growth factors. Although tumor formation is generally initiated by oncogenic mutations, growth factors are major regulators of tumor progression, including clonal expansion, invasion across tissue barriers, angiogenesis, and colonization of distant niches [83]. Growth factors can be secreted by other cell types from the tumor microenvironment, are released upon proteolytic degradation of the extracellular matrix, or result from autocrine production by the cancer cells themselves. In addition, growth factor receptor signaling can result from the presence of activating receptor mutations.

A study in EGF-stimulated cells identified that AKT1 promoted SRPK1 and SRPK2 autophosphorylation and translocation to the nucleus, with corresponding changes in alternative splicing of an *E1A* minigene reporter. Under these conditions, at least 398 changes in alternative splicing of endogenous transcripts were determined and of these about 75% were mediated by SRPK1 and SRPK2 [48,84]. Growth factor stimulation of mouse mammary epithelial cells also activated the PI3K/AKT pathway to regulate SRSF7 and SRSF1 levels, and promoted inclusion of the fibronectin ED-I exon [47], which increases the ability to promote cell cycle progression and wound healing. A similar regulation was found for Casp-9 in lung cancer cells in which activated AKT phosphorylates SRSF1, thereby inhibiting synthesis of anti-apoptotic Casp-9b isoform [52]. 

Hepatocyte growth factor (HGF) enhanced alternative splicing of a tumor-promoting variant of the tumor suppressor KLF6 by decreasing the levels of SRSF3 and, in consequence, also the levels of SRSF1 because the lack of SRSF3 triggers an alternative splicing into a non-functional *SRSF1* transcript degraded through the nonsense-mediated mRNA decay pathway [85].

Signaling through transforming growth factor β (TGF-β) during tumor progression causes epithelial-mesenchymal transition and is causally linked to metastasis. TGF-β was shown to induce widespread alterations in splicing affecting over 3600 genes. One major mechanism involved the transcriptional downregulation of the epithelial splicing regulatory proteins (ESRPs) through zinc finger E-box binding homeobox 1 (ZEB1) and ZEB2 transcriptional suppressor proteins [86]. The ESRPs regulate a network of transcripts that switch splicing during the epithelial-mesenchymal transition and have roles such as organization of actin cytoskeleton, cell to cell adhesion, cell polarity, and migration [87]. Reported examples were CD44, fibroblast growth factor receptor (FGFR), p120 catenin (CTNND1) [86] and RAC1b [88]. 

Interestingly, the TGF-β receptor I (TβRI) undergoes ligand-dependent nuclear translocation in cancer cells and forms a complex with several nuclear proteins implicated in posttranscriptional RNA processing, including splicing regulator hnRNPA1 [89]. This specifically induced alternative splicing events, including the generation of a soluble Epidermal growth factor receptor (EGFR) isoform.

The Insulin Growth Factor (IGF) receptor activates SRPK1 to switch between two VEGF splicing isoforms in regulating the balance of pro- and anti-angiogenic VEGF isoforms [75,90]. The same receptor can also activate the RAS-ERK signaling pathway and upregulate *INSR* (insulin receptor) exon 11 inclusion through the splicing factor SRSF1 in pancreatic β cells [91], or induces alternative splicing of *PKCβII* mRNA in L6 cells via the PI3K signaling pathway and phosphorylation state of SRSF5 [92].

In colorectal cancer cells, soluble factors of epithelial origin were shown to induce a constitutively active splice variant of the macrophage stimulating 1 receptor (*MST1R*) gene encoding the receptor tyrosine kinase named RON [40]. The pathway involves activation of ERK to phosphorylate SAM68, which then promotes stable transcript levels of *SRSF1*, the splice-enhancing factor that promotes skipping of *RON* exon 11. This change in SRSF1 expression counteracts the antagonistic binding of hnRNPA1 to a nearby silencer element [93] and favors the generation of the active RON variant with signaling properties promoting the epithelial-mesenchymal transition [94]. 

#### 3.1.2. Immune Cell or Inflammation-Derived Signals

The tumor microenvironment generally contains immune cells that release cytokines and these are perceived both, by other immune cells and tumor cells of epithelial origin. The corresponding effects on alternative splicing have yet been poorly explored. In one study, breast epithelial cells responded to the release of interferon γ (IFN-γ) by immune cells with Janus Kinase/Signal transducer and activator of transcription (JAK/STAT) pathway-mediated induction of Interferon regulatory factor-1 (IRF-1) expression. IRF-1 is a master regulator of IFN-γ-induced gene transcription, but transcriptome sequencing revealed that IRF-1 also affected alternative splicing of various genes involved in the regulation of growth and differentiation. For example, the carcinoembryonic antigen-related cell adhesion molecule-1 (CEACAM1) generates a long and pro-invasive variant whenever hnRNP proteins bound to variable exon 7 can form a complex with promoter-bound IRF-1 [95].

IFN-γ and interleukin (IL)-1b also led to important changes in alternative splicing in pancreatic β cells by modulating splicing in about 20% of all expressed genes [96]. Similarly, IL-6 or granulocyte-macrophage colony stimulating factor (GM-CSF) modulated alternative splicing of *BCL2L1* in K562 leukemia cells towards its anti-apoptotic splice variant BCL-x(L). Both cytokines required different intronic sequences for their responses, but the underlying molecular mechanisms remained unclear [97]. It was undetermined as to which mechanism is used by cytokines to induce the observed alternative splicing of induced nitric oxide synthase (*iNOS*) in d-lactate dehydrogenase (DLD1) and A549 tumor cells [98].

It is interesting to note that the use of non-steroidal anti-inflammatory drugs can also influence alternative splicing. Treatment of colon cancer cells with ibuprofen, for example, led to inhibition of tumor-related RAC1b [99] and this involved reduced phosphorylation of SRSF1, which is required for inclusion of the alternative exon [100]. 

### 3.2. Metabolic Stress Conditions

Tumor cells respond to adverse conditions with the activation of stress signaling pathways and adapt their gene expression program, including changes in alternative splicing.

#### 3.2.1. Hypoxia 

Cancer cells are often confronted with a significant reduction in oxygen availability, which is a major factor of selective pressure on tumor cell survival. Hypoxic regions have been identified within all solid tumors and their presence has been linked to malignant progression, metastasis, resistance to therapy, and poor clinical outcomes following treatment. Cellular responses to hypoxia are mediated by hypoxia inducible transcription factors (HIFs) [101]. In hepatocellular carcinoma cells, cultivated under hypoxia-mimicking conditions, exon array analysis showed 3059 alternative splicing events in 2005 genes [102]. The HIF activation can act through increased expression of CLK1 kinase leading to global hyperphosphorylation of SR proteins and the activation of hypoxia-dependent splice sites in HeLa cells [103]. 

#### 3.2.2. Oxidative Stress

Excessive generation of reactive oxygen species (ROS) in the body interferes with signaling pathways and is involved in several pathological conditions including cancer [104]. In a human gastric cancer cell line (AGS), oxidative stress led to phosphorylation and translocation of splicing factor TRA2B from the nucleus to the cytoplasm. In consequence, alternative splicing of several variable exons in the invasiveness-related *CD44* gene was observed [105].

#### 3.2.3. Osmotic Stress

Stress signals emanating from osmotic shock activate the p38-MAPK pathway via upstream kinases mitogen-activated protein kinase (MKK3/6). Activation of the p38-MAPK pathway, but not mitogenic stimulation, induces hnRNPA1 phosphorylation in the nucleus and then export into the cytoplasm [106,107,108]. The stress-induced decrease in nuclear hnRNPA1 abundance changed the alternative splicing pattern of an adenovirus *E1A* pre-mRNA splicing reporter and is expected to affect many endogenous alternative splicing events. Another effect observed under osmotic stress is the translocation of SRPK1 to the nucleus following differential association with heat shock proteins [84], which suggests that stress signaling also triggered differential phosphorylation of SR proteins besides affecting cytoplasmic translocation of hnRNPA1.

#### 3.2.4. Genotoxic Stress

DNA damage leads to genotoxic stress and can be caused by ROS, alkylating chemicals, irradiation or certain anti-cancer drugs. Following ultra violet (UV) irradiation or cisplatin treatment, for example, the stress-inducible alternative splice forms of mouse double minute 2 homolog (MDM2), MDM2-ALT1 and MDMX-ALT2, lack the p53-binding domain, and were detected in breast cancer cells as important modifiers of the p53-mediated cellular stress response [109]. 

Cisplatin treatment also led to phosphorylation of splicing factor SRSF2 but prevented its acetylation, a novel post-translational modification leading to SRSF2 protein accumulation. The acetyltransferase Tip60 acetylates SRSF2 on lysine 52 within the RNA recognition motif, and this triggered proteasomal degradation, whereas deacetylase histone deacetylase 6 (HDAC6) reversed acetylation as a positive regulator of SRSF2 protein levels. In addition, Tip60 inhibited SRSF2 phosphorylation by modulating the nuclear translocation of both SRPK1 and SRPK2 kinases. As a target transcript in response to cisplatin treatment, alternative splicing of an anti-apoptotic caspase-8 variant was identified that determined whether cells underwent apoptosis or G2/M cell cycle arrest [110,111]. 

Recently, a pathway linking DNA damage repair to the control of alternative splicing regulation was defined in keratinocytes. Ultra-violet irradiation induces cyclobutane pyrimidine dimers and the nucleotide excision repair pathway activates the protein kinase ATR, which promotes RNA polymerase II hyperphosphorylation. This modification decreased the elongation rate of transcription, which then allows time for the binding of lower-affinity splicing regulators and results in alternative splicing, in this case, in a group of 170 genes involved in defining cell survival, cell cycle progression or DNA repair [112].

### 3.3. Extracellular Matrix-Derived Signals

The extracellular matrix (ECM) has an important structural support function for cells but is not a static entity. Tumor cells or stromal cell types including fibroblasts can modulate the ECM in response to wounding, inflammation or cancer cell-derived stimuli. Changes in matrix composition, 3D-organization or matrix stiffness communicate with a diversity of cell surface receptors [113,114] and result in a signaling response [115], including changes in alternative splicing.

In mammary epithelial cells, a laminin-rich basement membrane activated mostly α6β4 integrin receptors that stimulate c-Jun N-terminal kinase (JNK) signaling. As a result, alternative splicing of the fibronectin gene at the ED-I and ED-III regions is inhibited [116]. By contrast, on stiff ECM substrates, β1-containing integrin complexes stimulate PI3K-AKT signaling, leading to the activation of the SR proteins that promote the inclusion of the cell cycle promoting ED-I exon region [117]. In parallel, this pathway affected further splicing events, leading to the production of PKC βII and the anti-angiogenic VEGF165b splice variants. Thus, depending on the ECM composition and the type of integrins activated, different intracellular signaling pathways mediate antagonistic splicing decisions. 

Finally, remodeling the ECM through experimental activation of extracellular matrix metalloproteinase 3 (MMP-3) in mouse mammary epithelial cells induced the expression of splice variant RAC1b, primarily through release of the repressor hnRNPA1 from the alternative exon [118]. In these cells, RAC1b caused an increase in cellular ROS and stimulated the expression of the transcription factor Snail, which induced epithelial-mesenchymal transition [119].

## 4. Concluding Remarks

Aberrant profiles observed in tumors mostly reflect the selection of endogenous alternative splicing variants with different functional properties that allow the malignant progression of initiated tumor cells and thus contribute specifically to tumor progression. 

**Figure 1:**
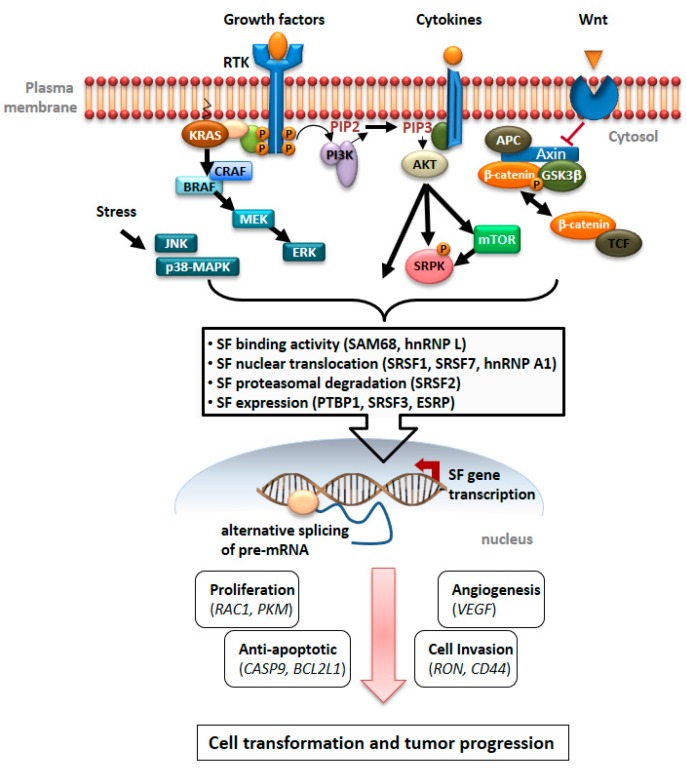
Schematic representation of the important connections between cancer cell signaling and the regulation of alternative splicing that the manuscript summarizes. Shown are the key components of the receptor tyrosine kinase (RTK), activated rat sarcoma viral oncogene homolog (RAS), rapidly accelerated fibrosarcoma (RAF)/extracellular signal-regulated kinase (ERK) and phosphatidylinositol 3 kinase (PI3K)/ v-akt murine thymoma viral oncogene homolog (AKT) pathways as well as the wingless (Wnt) pathway. Their activation can occur by the several oncogenic mechanisms described in the manuscript and lead to the modulation of splicing factor (SF) activity, localization or expression levels, as exemplified. These modifications have consequences for the choice of alternative exons during pre-mRNA splicing in the nucleus and cause the expression of variant proteins with distinct functional properties. These variants provide selective advantage for tumor cell progression and some of the examples described in the manuscript are mentioned.

Figure 1 schematically summarizes the mechanisms by which deregulated cancer cell signaling affects the molecular decisions of the splicing machinery. Much remains to be learned about these mechanisms, but also to what extent mutational events that promote the generation of certain alternative splicing variants may shape cancer cell signaling. 

In this respect, the study of individual splicing variants is important for several reasons: it helps with understanding of the basic principles underlying splicing regulation, and is an opportunity to identify variant proteins as novel biomarkers, or even therapeutic targets in specific tumor types. Examples for therapeutic options are the development of small molecule inhibitors that target the function or activity of a tumor-promoting variant, or the downregulation of its expression using RNA interference strategies or splice-switching anti-sense oligonucleotides. In addition, spliceosome inhibitory drugs such as Spliceostatin A or Sudemycins, which target the U2 snRNP component SF3B1 [120], revealed some tumor cell specific cytotoxic effects in leukemia that were associated with specific changes in alternative splicing [121]. However, it will be important to move our understanding from individual splicing variants to the overall pattern of alternative splicing observed in a given cancer cell. The reason is that whenever a splicing factor suffers changes in its expression level, activity, or subcellular location, then a plethora of splicing decisions in many different genes will be affected in the same cell. In the case of SRSF1, for example, its selective depletion in cells was reported to affect 498 splicing events [122]. 

In addition, fine-tuning of alternative splicing patterns arises from a combinatorial mode of splicing regulation, based on competition or collaboration between different splicing factors or RNA-binding proteins. The complexity in understanding fine-tuning is reflected by the fact that bioinformatic work identified 254 proteins in the human genome with experimental evidence for their involvement in splicing [123]. In addition, a more recent biochemical approach identified 860 proteins in HeLa cells that are able to bind RNA, including over 300 proteins not previously recognized as RNA-binding [124]. Integrating this information with cell signaling events into a network of functionally relevant changes in a given gene expression program is a current major challenge.

## Supplementary Materials

The following material is available online at The following are available online at www.mdpi.com/2073-4425/9/1/9/s1. Table S1: Examples of cancer-associated alternative splicing variants and their function.
