# Supplementary material for: Signaling Pathways Driving Aberrant Splicing in Cancer Cells
Source: Genes (Basel). 2017 Dec 29;9(1):9. doi: 10.3390/genes9010009 (PMC5793162; doi:10.3390/genes9010009)
Supplement: Supplementary file 1 [file genes-09-00009-s001.docx]

**Table S1.** Examples of cancer-associated alternative splicing variants and their function.

| **Gene** | **Splice variant** | **Cancer type** | **Function of variant** | **Reference** |
| --- | --- | --- | --- | --- |
| *NUMB* | Inclusion of exon 9 | Colon cancer | Promotes cell proliferation | [36] |
| *PKM* | Inclusion of exon 10 and skipping of exon 9 | Colon cancer and others | Promotes aerobic glycolysis and anabolic metabolism | [37] |
| *CD44* | Inclusion of exon v5 | Lymphoma and others | Increases metastatic potential | [38-40] |
| *BCL2L1* | Alternative in-frame 5’ splice site in exon 2 | Leukemia | Inhibition of apoptosis | [43,97] |
| *FN1* | Exclusion of ED-II exon | Ovarian cancer and others | Promotes cell-cycle progression and wound healing | [47,115] |
| *CASP9* | Exclusion of a four-exon cassette | Non–small cell lung cancer | Lacks caspase activity and inhibits apoptosis | [52-54] |
| *KLF6* | Alternative 5’ splice site in exon 2 | Hepatocellular carcinoma | Cytosolic retention of tumor-suppressing transcription factor | [57,85] |
| *PKC* | Inclusion of exon βII | Colon cancer | Different C-terminus, induces hyperproliferation | [60] |
| *RAC1* | Inclusion of in-frame exon 3b | Colorectal cancer | Stimulates NF-κB and sustains cell survival in cooperation with BRAF-V600E | [63] |
|  |  | Breast cancer | Promotes reactive oxygen species and epithelial-mesenchymal transition | [118] |
| *SLC39A14* | Inclusion of exon 4B and skipping of exon 4A | Colorectal cancer | Increased metal ion uptake | [66] |
| *VEGF* | Alternative 3’ splice site in exon 8 | Sarcoma, melanoma, prostate, renal, and colon cancer | Alternative C-terminus blocks angiogenic effect of VEGF | [75,90] |
| *MST1R (RON)* | Skipping of exon 11 | Colon and gastric cancer | Activated receptor version promoting epithelial-to-mesenchymal transition | [93,94] |
| *CASP8* | Retained intron | Breast cancer | Lacks caspase activity and inhibits apoptosis | [110,111] |

The listed genes are: Endocytic adaptor protein (*NUMB*), pyruvate kinase (*PKM*), clusters of differentiation 44 (*CD44*), B-cell CLL/lymphoma 2-like 1 (*BCL2L1*), Fibronectin (*FN1*), Caspase 9 (*Casp-9*), Krüppel-like factor 6 (*KLF6*), protein kinase C (*PKC*), Ras-related C3 botulinum toxin substrate 1 (*RAC1*), solute carrier family 39 (zinc transporter) member 14 (*SLC39A14*), vascular endothelial growth factor (*VEGF*), macrophage stimulating 1 receptor *MST1R* (*RON*), caspase 8 (*Casp-8*)
